# Antioxidant Function and Metabolomics Study in Mice after Dietary Supplementation with Methionine

**DOI:** 10.1155/2020/9494528

**Published:** 2020-10-20

**Authors:** Manrong Yu, Hui Chen, Pan Liu, Mei Yang, Leqin Zou, Dingfu Xiao

**Affiliations:** ^1^College of Animal Science and Technology, Hunan Agricultural University, Changsha Hunan 410128, China; ^2^Hunan Collaborative Innovation Center of Animal Production Safety, Changsha Hunan 410128, China

## Abstract

The antioxidant function and metabolic profiles in mice after dietary supplementation with methionine were investigated. The results showed that methionine supplementation enhanced liver GSH-Px activity and upregulated Gpx1 expression in the liver and SOD1 and Gpx4 expressions in the jejunum. Nrf2/Keap1 is involved in oxidative stress, and the western blotting data exhibited that dietary methionine markedly increased Keap1 abundance, while failed to influence the Nrf2 signal. Metabolomics investigation showed that methionine administration increased 2-hydroxypyridine, salicin, and asparagine and reduced D-Talose, maltose, aminoisobutyric acid, and inosine 5'-monophosphate in the liver, which are widely reported to involve in oxidative stress, lipid metabolism, and nucleotides generation. In conclusion, our study provides insights into antioxidant function and liver metabolic profiles in response to dietary supplementation with methionine.

## 1. Introduction

Methionine is an essential amino acid and mainly contributes to protein and S-adenosylmethionine (SAM) synthesis in the liver. SAM serves as a versatile methyl donor for nucleic acids and proteins, and the methylation of DNA and proteins is a major regulating mechanism for various physiological functions, including heat shock response [1, 2], regulation of intestinal barrier integrity [3], development [4], and gene expression [5]. Meanwhile, methionine has been widely demonstrated to involve in oxidative stress via the transsulfuration pathway for cysteine generation [6]. Cysteine further corresponds for glutathione (GSH) production [6], which is a major thiol group and contributes to antioxidant function against free radical species [7–10].

Here, we found that methionine enhanced antioxidant function via increasing antioxidant enzyme activity and expression. In addition, gas chromatography-mass spectrometry (GC-MS) was performed to investigate the liver metabolic profile after dietary methionine, and the results showed that 7 metabolites (i.e., hydroxypyridine, salicin, asparagine, D-talose, maltose, aminoisobutyric acid, and inosine 5'-monophosphate) were altered and these metabolites are involved in oxidative stress, lipid metabolism, and nucleotides generation.

## 2. Methods and Materials

### 2.1. Animal and Group

Twenty female ICR mice weighting 22.75 ± 0.43 g were assigned into two experimental groups: a control group in which mice received a normal diet (containing 0.43% methionine) and a Met group in which mice received an additional 0.5% DL-methionine [11]. Body weight and feed intake were recorded. After three weeks, blood was collected from the eyes, liver, and jejuna, and ilea samples were collected. This study involving animal subjects was approved by the College of Animal Science and Technology, Hunan Agricultural University.

### 2.2. Antioxidant Function

Serum samples were separated and stored for further analyses. Liver samples were homogenized; then, the supernatant was collected for further analyses. Antioxidant indexes (i.e., superoxide dismutase (SOD), glutathione peroxidase (GSH-Px), and catalase (CAT)) were tested (Nanjing Jiancheng, China) [12].

### 2.3. RT-PCR

RNA isolation and cDNA synthesis were conducted according to previous studies [13]. Primer 5.0 was used to design the primer (Table 1). RT-PCR was performed according to previous studies [14–18].

### 2.4. Western Blot

Liver proteins were extracted by the Thermo Fisher kits (Waltham, MA, USA) and separated by SDS-PAGE electrophoresis [14, 19, 20]. Then, the separated proteins were transferred onto PVDF membranes (Millipore, MA, USA) for the incubation of antibodies, including taste receptor type 1 member 1 (T1R1), taste receptor type 1 member 3 (T1R3), Kelch-like ECH-associating protein 1 (Keap1), nuclear factor erythroid *2*-related factor *2* (Nrf2), and uncoupling protein 2 (Ucp2) (Abcam Bio).

### 2.5. Gas Chromatography-Mass Spectrometry (GC-MS) Analysis

Metabolomics study of liver samples was investigated (Agilent 7890A, Agilent, USA), and data were analyzed by the Chroma TOF 4.3X software (LECO Corporation, USA) and LECO-Fiehn Rtx5 database [21–25].

### 2.6. Statistical Analysis

All data were analyzed using the Students' *T* test (SPSS 17.0 software). Data are expressed as the mean ± sem. Values in the same row with different superscripts are significant (*P* < 0.05).

## 3. Results

### 3.1. Growth Performance

Dietary 0.5% methionine had no effect on the body weight gain, average feed intake, and the ratio of feed intake to body weight gain (*P* < 0.05) (Table 2).

### 3.2. Antioxidant Function

Serum and liver GSH-Px, SOD, and CAT were measured to evaluate the antioxidant function after dietary supplementation with methionine (Table 3). In the serum, we did not find any significant difference on GSH-Px, SOD, and CAT activities (*P* > 0.05). In addition, methionine supplementation markedly enhanced liver GSH-Px activity (*P* < 0.05), while liver CAT activity was inhibited in the Met group (*P* < 0.05).

**Figure 1:**
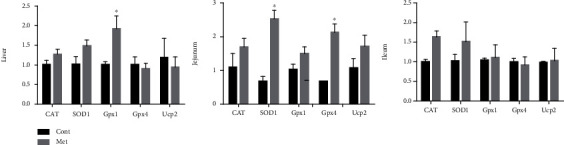
The expression of antioxidant genes (CAT, SOD1, Gpx, Gpx4, and UCP2) in the liver, jejunum, and ileum.

We further determined the expression of antioxidant genes (CAT, SOD1, Gpx, Gpx4, and UCP2) in the liver, jejunum, and ileum (Figure 1). In the liver, methionine markedly upregulated the Gpx1 expression (*P* < 0.05). In the jejunum, methionine markedly increased SOD1 and Gpx4 mRNA abundances (*P* < 0.05).

### 3.3. T1R1 and T1R3

**Figure 2:**
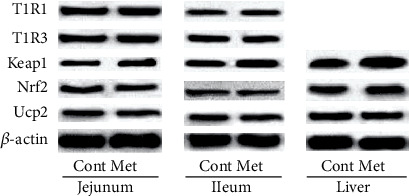
Western blot: T1R1 and T1R3 of jejunum, ileum, and liver.

Intestinal T1R1 and T1R3 were determined via western blot (Figure 2). In the jejunum and ileum, dietary supplementation failed to influence T1R1 and T1R3 abundance (*P* > 0.05).

### 3.4. Nrf2/Keap1 Signal

In this study, dietary supplementation significantly enhanced the liver and ileal Keap1 expression (*P* < 0.05), but Nrf2 expression was not changed (*P* > 0.05).

### 3.5. Metabolic Profiles Analyzed by GC-MS

**Figure 3:**
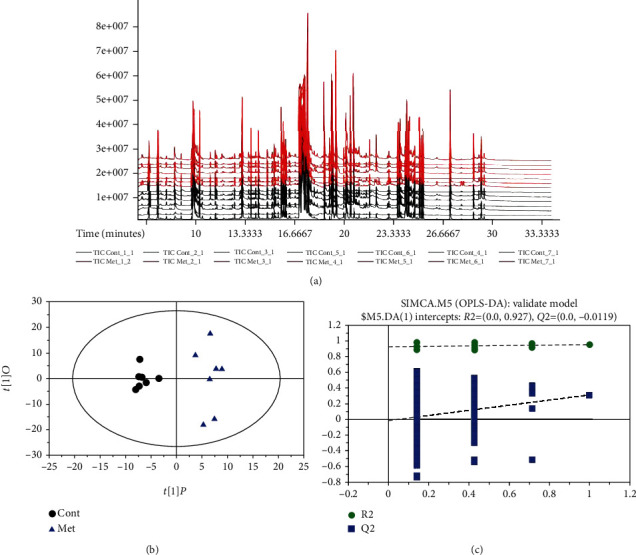
Metabolites identified from 534 peaks in the chromatograms and PLS-DA score plots.

Metabolites in the liver were analyzed from 534 peaks (Figure 3(a)). PLS-DA score plots exhibited that dietary supplementation with methionine leaded distinctive metabolic profiles in mice compared with the control mice (Figure 3(b) and 3(c)).

The identified potential markers after dietary supplementation with methionine are listed in Table 4. Dietary methionine significantly increased liver 2-hydroxypyridine, salicin, and asparagine concentrations. Meanwhile, liver D-talose, maltose, aminoisobutyric acid, and inosine 5'-monophosphate were markedly reduced after dietary methionine.

## 4. Discussion

Methionine is a limiting AA in most cereal soybean-based diets for growing pigs [26]. Recently, methionine has been reported to involve in lipid metabolism [27], translational capacity [28], autophagy [29], and maintenance and differentiation of pluripotent stem cells [30]. Meanwhile, dietary methionine or methionine restriction has been demonstrated to influence growing performance [31, 32], while the current study did not observed any significant difference in the growth performance in mice. In addition, we found that dietary supplementation with methionine enhanced antioxidant function and upregulated liver Keap1 expression. Meanwhile, 7 metabolites were found related to dietary methionine based on GC-MS-based metabolomics investigation.

Methionine can sustain cellular redox homeostasis via the generation of cysteine, which is a substance for GSH synthesis [6, 33]. In this study, we found that dietary methionine increased antioxidant function evidenced by the enhanced serum GSH-Px activity and upregulation of Gpx1, Gpx4, and SOD1. Methionine can form cysteine via the transsulfuration pathway to serve as a precursor for GSH, hydrogen sulfide, and taurine [6]. These metabolites have been demonstrated to mediate oxidative stress via scavenging hydroxyl radical and superoxide directly and serving as a cofactor for the antioxidant enzymes, including GSH peroxidase (Gpx) [6]. Meanwhile, Campbell et al. reported that methionine affects the oxidative branch of the pentose phosphate pathway and increased abundance of the NADPH producing enzyme 6PGDH, which further plays a protective role against oxidative stress [34].

Nrf2/keap1 signaling pathway is widely involved in oxidative stress and regulates the expression of antioxidant genes [12, 14, 15]. Normally, Nrf2 protein is sequestered in the cytosol via targeting Keap1 and soon be degraded under cellular homeostasis [35]. Although Keap1 was upregulated after dietary methionine, liver, jejunal, and ileal Nrf2 expression was not changed in this study. Nrf2 activation was observed under methionine restriction [36, 37], which further regulated antioxidant genes and oxidative stress.

Metabolomics investigation revealed 7 metabolites after dietary methionine, including hydroxypyridine, salicin, asparagine, D-Talose, maltose, aminoisobutyric acid, and inosine 5'-monophosphate. Salicin has been studied as a potent anti-inflammatory agent and exhibits an anti-inflammatory and antioxidant functions in various inflammation-related diseases [38, 39]. Asparagine is a metabolic product of the aspartate, which has been demonstrated to alleviate diquat-induced oxidative injury in piglets [40]. Thus, the increased contents of salicin and asparagine in the Met group may indicate methionine mediates inflammation and oxidative stress via indirectly affecting salicin and asparagine concentrations. Aminoisobutyric acid, a low molecular myokine, contributes to the conversion of white adipose tissue into brown fat, accelerating the breakdown of lipids into heat, water, and CO_2_ [41]. Previous reports also demonstrated that methionine regulates lipid metabolism via increasing metabolic flexibility and overall insulin sensitivity [42]. Inosine 5'-monophosphate is an intermediate of purine metabolism and serves as a precursor for other nucleotides used for metabolic functions [43, 44]. Thus, we speculated that dietary methionine serves as a regulator in nucleotide metabolism, which was further demonstrated by Benjamin P. Tu who reported that methionine regulates translational capacity through modulation of tRNA thiolation [28].

In conclusion, dietary supplementation with methionine enhanced antioxidant function. Meanwhile, metabolomics investigation revealed that methionine influenced 7 metabolites, including hydroxypyridine, salicin, asparagine, D-Talose, maltose, aminoisobutyric acid, and inosine 5'-monophosphate.

## Figures and Tables

**Table 1:** PCR primer sequences: the forward primers (F) and the reverse primers (R) used in this study.

Gene	Accession no.	Nucleotide sequence of primers (5′–3′)	Size (bp)
*β*-Actin	NM_007393.3	F:GTCCACCTTCCAGCAGATGT R:GAAAGGGTGTAAAACGCAGC	117
CAT	XM_006498624.1	F:AATATCGTGGGTGACCTCAA R:CAGATGAAGCAGTGGAAGGA	243
ZnCuSOD	NM_011434.1	F:CCACTGCAGGACCTCATTTT R:CACCTTTGCCCAAGTCATCT	216
Gpx1	NM_008160.6	F:GGTTCGAGCCCAATTTTACA R:CCCACCAGGAACTTCTCAAA	199
Gpx4	NM_001037741.3	F:CTCCATGCACGAATTCTCAG R:ACGTCAGTTTTGCCTCATTG	117
UCP2	NM_011671.5	F:TAGTGCGCACCGCAGCC R:AGCTCATCTGGCGCTGCAG	126

**Table 2:** Growth performance after dietary supplementation with methionine. IBD: initial body weight; FBW: final body weight; AFI: average feed intake; ABWG: average body weight gain; F : G: the ratio of feed intake to weight gain.

Item	IBW	FBW	AFI	ABWG	F : G
Cont	22.26 ± 0.37	28.27 ± 0.53	5.65 ± 0.05	0.30 ± 0.01	19.36 ± 0.88
Met	23.24 ± 0.50	26.84 ± 0.47	5.76 ± 0.02	0.27 ± 0.02	21.85 ± 1.26

**Table 3:** GSH-Px, SOD, and CAT activities in the serum and liver after dietary supplementation with methionine. The ^∗^ means the difference is significant between the two groups (*P* < 0.05).

Item	GSH-Px	SOD	CAT
Serum (U/ml)			
Cont	17.89 ± 1.22	116.74 ± 20.48	366.52 ± 135.33
Met	28.95 ± 7.78	131.06 ± 12.51	216.77 ± 34.27
Liver (U/mgprot)			
Cont	26.80 ± 2.35	2342.63 ± 50.94	188.12 ± 15.24
Met	152.88 ± 30.01^∗^	2398.21 ± 70.69	116.08 ± 20.47^∗^

**Table 4:** List of differential metabolites between the control and Met groups.

ID	Peak	R.T.	Count	Mass	Cont	Met	VIP	*P* value	Fold change
13	2-hydroxypyridine	6.61	29	152	0.64	1.32	1.94	0.03	2.05
421	D-Talose	17.38	16	235	0.25	0.00	1.92	0.04	0.00
662	Maltose	24.22	17	361	4.39	1.10	1.96	0.03	0.25
204	Aminoisobutyric acid	12.31	23	174	0.11	0.04	2.04	0.04	0.36
597	Salicin	22.42	29	169	0.02	0.03	1.99	0.04	2.08
711	Inosine 5'-monophosphate	25.87	19	169	0.01	0.00	1.94	<0.01	0.09
300	Asparagine	14.37	19	229	0.00	0.01	2.69	<0.01	3980383.06

## Data Availability

The data used to support the findings of this study are available from the corresponding author upon request.
